# Synthesis and Analgesic Effects of μ-TRTX-Hhn1b on Models of Inflammatory and Neuropathic Pain

**DOI:** 10.3390/toxins6082363

**Published:** 2014-08-13

**Authors:** Yu Liu, Jianguang Tang, Yunxiao Zhang, Xiaohong Xun, Dongfang Tang, Dezheng Peng, Jianming Yi, Zhonghua Liu, Xiaoliu Shi

**Affiliations:** 1 College of Chemistry and Chemical Engineering, Hunan Institute of Science and Technology, Yueyang 414006, Hunan, China; E-Mails: hiliu2000@126.com (Y.L.); xiaohongxun2012@126.com (X.X.); tdfyouxiang@126.com (D.T.); pdz29167641@163.com (D.P.); yjm91@163.com (J.Y.); 2 The Second Xiangya Hospital of Central South University, Changsha 410008, Hunan, China; E-Mail: tjg168@aliyun.com; 3 College of Life Sciences, Hunan Normal University, Changsha 410081, Hunan, China; E-Mail: yunxiao_zhang@126.com

**Keywords:** μ-TRTX-Hhn1b, Nav1.7, analgesic, inflammatory pain, neuropathic pain

## Abstract

μ-TRTX-Hhn1b (HNTX-IV) is a 35-amino acid peptide isolated from the venom of the spider, *Ornithoctonus hainana*. It inhibits voltage-gated sodium channel Nav1.7, which has been considered as a therapeutic target for pain. The goal of the present study is to elucidate the analgesic effects of synthetic μ-TRTX-Hhn1b on animal models of pain. The peptide was first synthesized and then successfully refolded/oxidized. The synthetic peptide had the same inhibitory effect on human Nav1.7 current transiently expressed in HEK 293 cells as the native toxin. Furthermore, the analgesic potentials of the synthetic peptide were examined on models of inflammatory pain and neuropathic pain. μ-TRTX-Hhn1b produced an efficient reversal of acute nociceptive pain in the abdominal constriction model, and significantly reduced the pain scores over the 40-min period in the formalin model. The efficiency of μ-TRTX-Hhn1b on both models was equivalent to that of morphine. In the spinal nerve model, the reversal effect of μ-TRTX-Hhn1b on allodynia was longer and higher than mexiletine. These results demonstrated that μ-TRTX-Hhn1b efficiently alleviated acute inflammatory pain and chronic neuropathic pain in animals and provided an attractive template for further clinical analgesic drug design.

## 1. Introduction

Voltage-gated sodium channels (VGSCs) are essential for the initiation and propagation of action potentials in excitable tissues, such as nerves and muscles. Considerable *in vivo* and *in vitro* studies indicate that VGSCs play a key role in neuropathic and inflammatory pain, which begins with the aberrant firing of action potential bursts in damaged neuronal tissue [1,2,3,4]. Of the nine subtypes of VGSCs identified to date and designated as Nav1.1–1.9, Nav1.3, 1.7, 1.8 and 1.9 play specialized roles in nociceptive pathways and are considered ideal targets for the development of analgesic drugs [4,5,6,7]. Compelling genetic evidence linking Nav1.7 to pain in human beings has been provided by the identification of dominant gain-of-function and recessive loss-of-function mutations in *SCN9A*, the gene that encodes Nav1.7 [8,9,10]. Dominant gain-of-function mutations of Nav1.7 have been linked to two severe pain syndromes, paroxysmal extreme pain disorder (PEPD) and inherited erythromelalgia (IEM). PEPD is well controlled by the VGSCs blocker, carbamazepine [9,11,12]. Treatment for IEM, even with VGSCs blockers, e.g., lidocaine or mexiletine, is very ineffective [8,13] and, in one case, may be the result of the decreased affinity of the mutant channel to small molecular blocker drugs [14]. Recessive loss-of-function mutations of Nav1.7 have been linked to congenital insensitivity to pain (CIP). Mutation of the *SCN9A* truncates the channel protein or impairs splicing signals to prevent the production of channel mRNA [10]. Truncated Nav1.7 mutant channels do not produce functional channels [10,15] or act as dominant negative proteins [15]. Patients do not report any form of pain, but report intact sensory modalities, except for impaired olfaction [16], and do not display motor, cognitive, sympathetic or gastrointestinal deficits. These studies provide strong evidence for the importance of Nav1.7 in pain therapy.

Peptide toxins, including µ-conotoxins, α-scorpion and β-scorpion toxins, sea anemone toxins, β-spider toxins and µ spider toxins, interact with VGSCs and either block Na^+^ currents or modulate the gating properties of these channels [17,18,19,20,21,22]. Certain peptide toxins bind to VGSCs with high affinity and selectivity, and some of them have been used to isolate VGSC subtypes and to explore their structure and function. Moreover, peptide toxins have been used to develop therapeutic drugs that selectively target certain VGSC subtypes [17,18,19,20,21,22].

μ-TRTX-Hhn1b (HNTX-IV) is a 35-residue peptide from tarantula *Ornithoctonus hainana* (*Haplopelma hainanum*) venom [23,24]. The amino acid sequence is ECLGFGKGCNPSNDQCCKSSNLVCSRKHRWCKYEI. The peptide amidated at its C-terminal has a high proportion of basic residues and is crosslinked by three disulfide bonds. The linkage pattern of disulfide bridges is Cys2-Cys17, Cys9-Cys24 and Cys16-Cys31. μ-TRTX-Hhn1b specifically inhibits the neuronal TTX-S VGSCs in rat dorsal root ganglion (DRG) cells [25]. It was found that four residues (Lys27, His28, Arg29 and Lys32) form a positively charged patch on the molecular surface and are critical for the inhibitory activity of μ-TRTX-Hhn1b [7,26]. The four residues might interact directly with the acidic residues in the DIIS3-S4 linker of TTX-S sodium channels and stabilize the DIIS4 voltage sensor. Our unpublished data [27] showed that native μ-TRTX-Hhn1b preferentially inhibited Nav1.2, Nav1.3 and Nav1.7 compared with Nav1.4 and Nav1.5; Nav1.7 was the most sensitive channel to μ-TRTX-Hhn1b (IC_50_ ~21 nM). Considering the critical role of Nav1.7 in pain pathways, we want to ask whether μ-TRTX-Hhn1b possesses an analgesic effect on both inflammatory and neuropathic pain models. In the present studies, the peptide was successfully synthesized, and its inhibitory activity was examined on human Nav1.7 expressed in HEK 293 cells. Additionally, its analgesic properties in models of inflammatory and neuropathic pain were further determined. The results suggested that μ-TRTX-Hhn1b was indeed an attractive template for further clinical analgesic drug design.

## 2. Results and Discussion

### 2.1. Peptide Synthesis, Folding and Characterization

**Figure 1:**
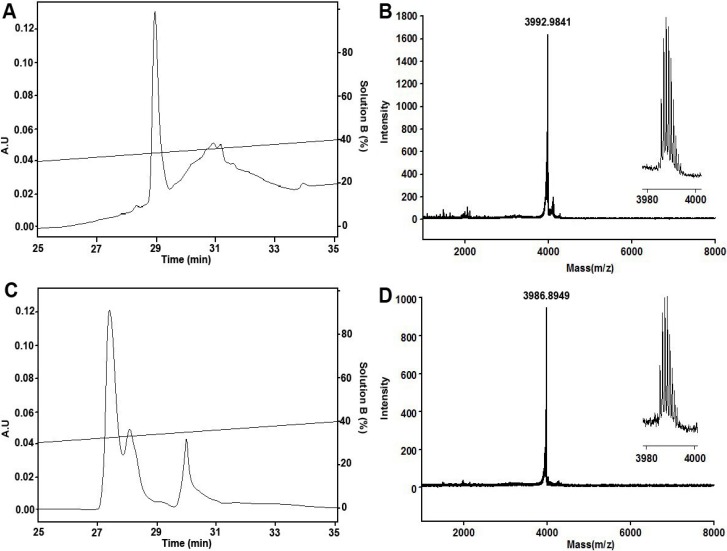
RP-HPLC purification and MALDI-TOF-MS of reduced and refolded μ-TRTX-Hhn1b. (**A**) Purification of linear reduced peptide; (**B**) MALDI-TOF-MS of linear reduced peptide; (**C**) purification of refolded/oxidized peptide; (**D**) MALDI-TOF-MS of refolded/oxidized peptide.

Solid-phase synthesis of μ-TRTX-Hhn1b, using Fmoc-protected amino acids and HOBt/TBTU coupling, yielded a major product, as revealed by reversed-phase high-performance liquid chromatography (RP-HPLC) analysis and matrix-assisted laser-desorption/ionization time-of-flight mass spectrometry (MALDI-TOF-MS) (Figure 1A,B). The measured monoisotopic molecular weight was 3992.9841 Da, which was consistent with that calculated from the linear reduced peptide amidated at its C-terminal. Then, the purified reduced peptide was refolded/oxidized in buffer 0.1 M Tris-HCl, 0.1 M NaCl, pH 7.8, containing 5 mM GSH and 0.5 mM GSSG for 24 h at room temperature. Purified product after oxidization was homogeneous in analytical RP-HPLC (Figure 1C), and the monoisotopic molecular weight was determined to be 3986.8949 Da (Figure 1D), 6 Da lower than that of the reduced peptide, indicating that the six cysteine residues form three pairs of disulfide bonds.

**Figure 2:**
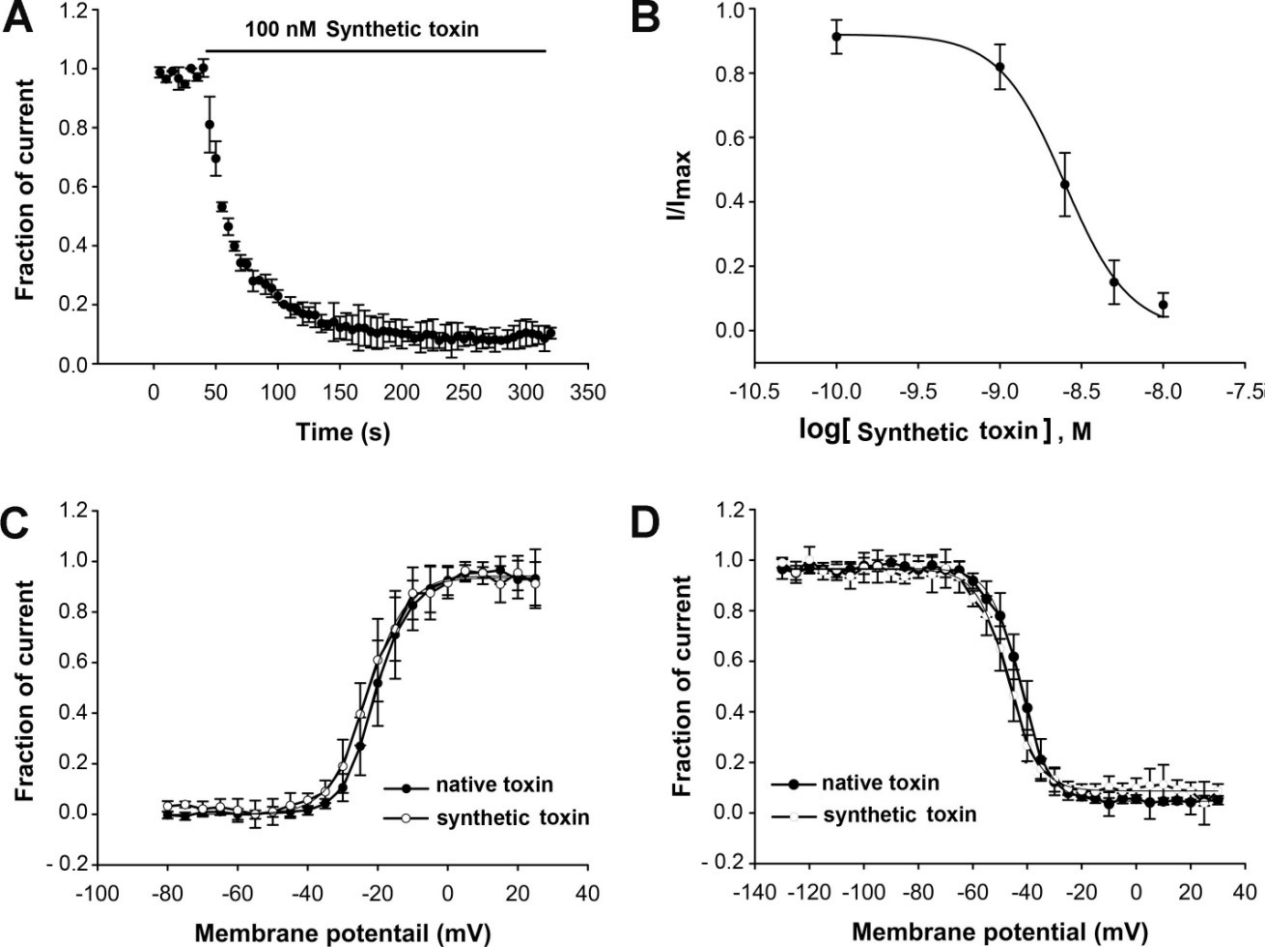
Inhibitory effect of synthetic μ-TRTX-Hhn1b on Nav1.7 expressed on HEK 293 cells. (**A**) Time course of inhibition of Nav1.7 by 100 nMμ-TRTX-Hhn1b (τ_on_ = 20.5 ± 0.3 s). (**B**) The inhibition of μ-TRTX-Hhn1b on Nav1.7 was dose-dependent. Each data point (mean ± S.E.), which was derived from five to eight cells, shows current relative to native toxin. Apparent IC_50_ values were approximately 21 nM; the inward Na^+^ currents were elicited by a 50-ms depolarization from a holding potential of −80 mV to −10 mV. μ-TRTX-Hhn1b did not significantly alter the activation (**C**) and steady-state inactivation (**D**) of Nav1.7. Data are plotted as a fraction of the maximum conductance. The voltage-dependence of steady-state inactivation was estimated using a standard double-pulse protocol, in which a 20-ms depolarizing test potential of 0 mV followed a 500-ms prepulse at potentials that ranged from −130 to −10 mV with a 10-mV increment. Cells were held at −100 mV.

Furthermore, we wanted to check if the synthetic μ-TRTX-Hhn1b exhibited the same inhibitory activity on VGSCs as the native peptide. The synthetic μ-TRTX-Hhn1b was therefore submitted to patch clamp analysis on human Nav1.7 transiently expressed in HEK 293 cells. The time-dependent inhibition of Nav1.7 by 100 nM μ-TRTX-Hhn1b (τ_on_ = 20.5 ± 0.3 s) was examined, as shown in Figure 2A. The inhibitory effect was dose-dependent (Figure 2B). The IC_50_ value was calculated to be approximately 21 nM. The effects of 50 nM μ-TRTX-Hhn1b on the activation and inactivation of Nav1.7 were analyzed. The half-activation voltage and half-inactivation voltage of Nav1.7 after treatment with 50 nM μ-TRTX-Hhn1b were −23.0 ± 0.4 mV and −47.1 ± 0.3 mV, respectively, compared to −20.5 ± 0.2 mV and −42.6 ± 0.2 mV, respectively, in the native toxin group (Figure 2C), indicating that μ-TRTX-Hhn1b inhibited the peak current of Nav1.7 without significant alteration of the activation and inactivation kinetics of Nav1.7. The results indicated that this peptide could be prepared by chemical synthesis instead of purification from the crude venom, and the synthetic peptide could be used for analgesic analysis, as described below.

### 2.2. The Analgesic Effect of μ-TRTX-Hhn1b on the Acetic Acid-Induced Abdominal Constriction Mouse Model

**Figure 3:**
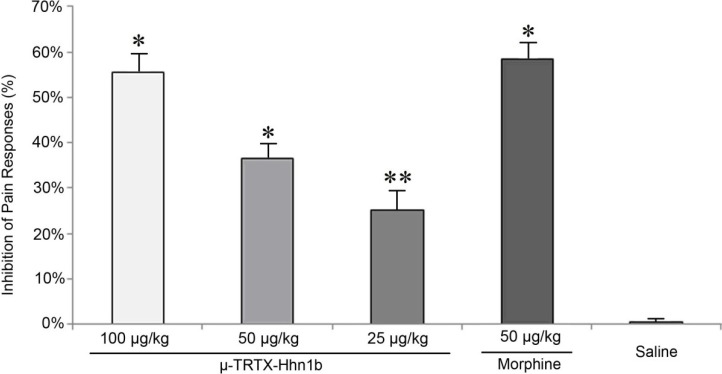
The analgesic effect of μ-TRTX-Hhn1b in the mouse abdominal constriction test. μ-TRTX-Hhn1b and morphine were administrated by i.p. injection 15 min before acetic acid application. Data are represented as the mean ± SEM of six animals per group. ** *p* < 0.05, * *p* < 0.01.

The acetic acid-induced constriction reaction in mice, described as a typical model for inflammatory pain, has long been used as a screening tool for the evaluation of analgesic or anti-inflammatory agents [28,29]. The major transmission pathway of this inflammatory pain has been documented as the pathway comprising peripheral polymodal receptors around small vessels that signal the central nervous system via sensory afferent C-fibers that enter the dorsal horn [30]. Bradykinin, substance P and prostaglandins [31,32] were regarded as mediators involved in the constriction responses induced by acetic acid. Intraperitoneal injection of acetic acid into mice generates an abdominal constriction response consisting of a wave of constriction and elongation passing caudally along the abdominal wall, sometimes accompanied by twisting of the trunk and followed by extension of the hind limbs [28]. As shown in Figure 3, μ-TRTX-Hhn1b signiﬁcantly reduced these pain responses evoked by acetic acid. The protective effect was dose dependent with a 25.2% (*p* < 0.05) reduction observed for 25.0 μg/kg μ-TRTX-Hhn1b and a 55.6% (*p* < 0.01) seen for 100 μg/kg μ-TRTX-Hhn1b, respectively. As a control, morphine at a dose of 50 μg/kg caused a 58.3% (*p* < 0.01) inhibition of pain responses, which is similar with the analgesic effects of 100 μg/kg μ-TRTX-Hhn1b.

### 2.3. The Analgesic Effect of μ-TRTX-Hhn1b on the Formalin-Induced Inflammatory Rat Model

**Figure 4:**
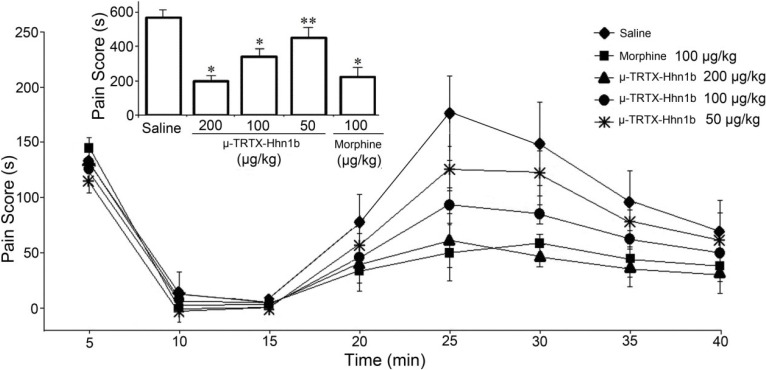
The analgesic effects of μ-TRTX-Hhn1b in the rat formalin test. μ-TRTX-Hhn1b and morphine were administrated by i.p. injection 15 min before formalin. The inset shows the sum of flinching, licking/biting in the formalin test during 40 min. Data are represented as the mean ± SEM of six animals per group. ** *p* < 0.05, * *p* < 0.01.

Formalin-induced paw licking is used to test the effects on peripheral pain, which typically resembles human clinical pain conditions [33,34]. The antinociceptive effects of μ-TRTX-Hhn1b on formalin-induced paw licking are illustrated in Figure 4. Responses in two time phases, including the early phase (0–5 min, non-inflammatory pain) and the late phase (15–30 min, inflammatory pain), are recorded, respectively. As shown in Figure 4, it was found that in contrast to i.p. saline injection, i.p. pre-treatment with μ-TRTX-Hhn1b (50, 100 or 200 µg/kg) resulted in a dose-dependent suppression of pain behaviors induced by s.c. formalin injection in the late-phase. The pain responses, including paw licking, lifting, favoring, shaking and flinching, were inhibited in the late phases, but not in the early phases. The column graph (Figure 4, inset) showed that the pain scores over the 40 min period in the μ-TRTX-Hhn1b-treated group at 50, 100 and 200 µg/kg doses were 458.5 ± 59.9 s (*n* = 6, *p* < 0.05), 346.0 ± 46.2 s (*n* = 6, *p* < 0.01) and 199.8 ± 32.6 s (*n* = 6, *p* < 0.01), respectively, while that in the control group (saline) was 569.2 ± 43.5 s (*n* = 6). In order to determine the antinociceptive efficacy of μ-TRTX-Hhn1b, morphine was used as a positive control. As shown in Figure 4, the pain score was 225.0 ± 55.8 s in 100 µg/kg morphine-treated group (*n* = 6, *p* < 0.01). The data indicated that μ-TRTX-Hhn1b at a dose of 200 µg/kg produced antinociceptive activity similar to 100 µg/kg of morphine.

The pain response induced by formalin in the late phase was significantly inhibited by μ-TRTX-Hhn1b, while μ-TRTX-Hhn1b had little effect on the pain responses induced by formalin in the early phase (Figure 4). The pain responses in the early phase are known as non-inflammatory, which is mediated by nociceptors in the paw and reflects central pain, while the pain response in the late phase is inflammatory attributed to prostaglandin (PG) synthesis [35]. The obviously antinociceptive function of μ-TRTX-Hhn1b in the late phase suggests that μ-TRTX-Hhn1b may attenuate peripheral pain associated with inflammation.

### 2.4. The Analgesic Effect of μ-TRTX-Hhn1b on Spinal Nerve Injury Rat Model

Several animal models of neuropathic pain were developed in the last 10–15 years, including the sciatic nerve chronic constriction injury model (CCI) [36] and the spinal nerve ligation model (SNL) [37]. The spared nerve injury (SNI) model is a new model of peripheral nerve damage recently developed by Decosterd and Woolf [38]. The SNI model differs from the CCI and SNL models in the location and form of injury. This model results in early, prolonged, robust (all animals are responders) behavioral modifications. The mechanical (von Frey hair) sensitivity and cold responsiveness increase in the ipsilateral sural nerve without any change in heat thermal thresholds. Furthermore, this model is reliable and easy to duplicate. These features may enable this new animal model to be a useful tool in screening new analgesic drugs. In the present study, we selected the SNI rat model to determine the analgesic effects of μ-TRTX-Hhn1b on neuropathic pain. SNI animals develop qualitative signs indicative of a marked sensory hypersensitivity of the ipsilateral hind paw to the nerve injury present 24 h after the surgery.

**Figure 5:**
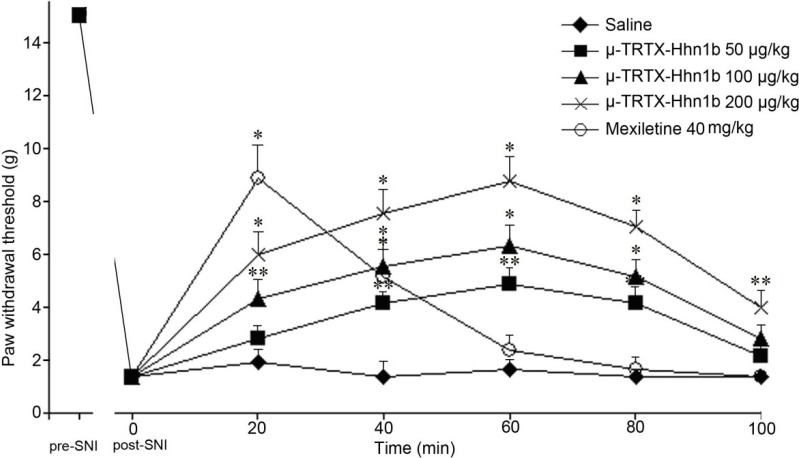
Effect of μ-TRTX-Hhn1b in the rat spared nerve injury (SNI)-induced allodynia model of neuropathic pain. The dose-response of the effect of μ-TRTX-Hhn1b was determined by using von Frey filaments to assess mechanical stimulation. μ-TRTX-Hhn1b significantly reversed SNI-induced allodynia at 50–200μg/kg. Data was represented as the mean ± SEM of six animals per group. ** *p* < 0.05, * *p* < 0.01.

Three days after surgery, significant mechanical allodynia would be developed on the injured hind paw. A total of 30 rats with mechanical allodynia were divided into five groups randomly. After intraperitoneal (i.p.) administration of different doses of μ-TRTX-Hhn1b (50 µg/kg, 100 µg/kg or 200 µg/kg), the mechanical allodynia induced by SNI was depressed in a dose-dependent manner (Figure 5). μ-TRTX-Hhn1b at the highest dose 200 μg/kg significantly increased the withdrawal threshold in response to von Frey hair stimulation 60 min after injection to 8.8 ± 0.9 g compared with saline (1.4 ± 0.4 g, *p* < 0.01). Even at 100 min after injection of μ-TRTX-Hhn1b, the withdrawal threshold remained 4.0 ± 0.6 g (*p* < 0.05). The reduction in the paw withdrawal response remained significantly different compared with the pre-injection baseline response throughout the entire testing period (Figure 5). For the treatment with mexiletine, i.p. injection of mexiletine at a dose of 40 mg/kg significantly increased the withdrawal threshold in response to von Frey hair stimulation 15 min after injection to 8.9 ± 1.2 g compared with vehicle and baseline (1.4 ± 0.4 g, *p* < 0.01); at 40 min after injection of mexiletine, the withdrawal threshold remained (5.1 ± 1 g, *p* < 0.01), before returning to a level non-significantly different from the baseline and vehicle at 60 min (Figure 5). Our results suggested that μ-TRTX-Hhn1b attenuated injury-induced nociceptive response. From Helle Kirstein Erichsen’s results [39], the administration of mexiletine (37.5 mg/kg, i.p.) significantly increased the withdrawal threshold in response to von Frey hair stimulation 15 min after injection, but 45 min after injection of mexiletine, the withdrawal threshold returned to a level non-significantly different from the baseline and vehicle, which was similar to our data showing that no analgesic effect was observed at 60 min after the application of mexiletine. However, it was interesting that the antinociceptive effect of μ-TRTX-Hhn1b could last for more than 100 min, about two-fold longer than that of mexiletine.

### 2.5. Rat Rotarod Model of Motor Coordination

**Figure 6:**
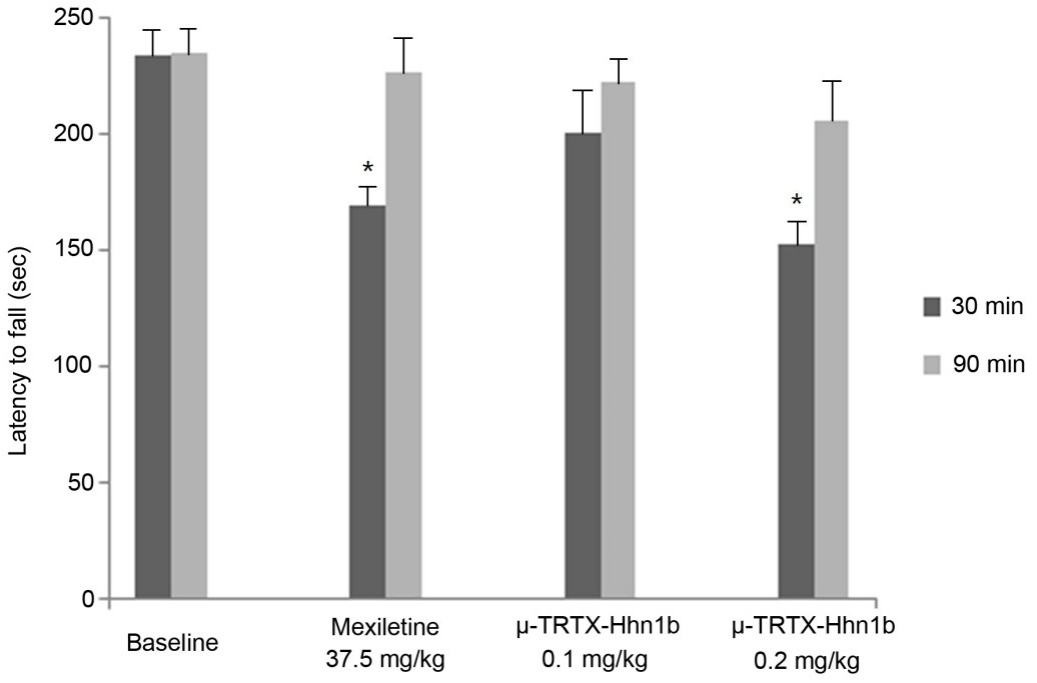
Motor side effects of μ-TRTX-Hhn1b were examined in rats by the rotarod latency test. At the beginning of the rotarod test, trained rats were tested 0 min before (baseline) and 30 and 90 min after μ-TRTX-Hhn1b or mexiletine administration. The latency to fall off the rotarod apparatus was determined from the mean time in three trials for each rat at each time. Data are represented as the mean ± SEM of six animals per group. * *p* < 0.05.

In the three tests described above, no animal death was observed, but μ-TRTX-Hhn1b at a dose of 0.2 mg/kg was found to cause some side effects, such as inactivity and paralysis. Therefore, the rotarod latency test was used to access the motor side effects of μ-TRTX-Hhn1b in rats. As shown in Figure 6, compared with baseline (0 min before the injection of μ-TRTX-Hhn1b), at a dose of 0.1 mg/kg, μ-TRTX-Hhn1b induced no significant change in latencies to fall off the rotarod at 30 min and 90 min after drug administration, while μ-TRTX-Hhn1b at a dose of 0.2 mg/kg significantly (*p* < 0.05) reduced latencies at 30 min after μ-TRTX-Hhn1b administration, but not at 90 min after μ-TRTX-Hhn1b administration; as the positive control drug, mexiletine at a dose of 37.5 mg/kg also led significant latency reduction at 30 min. A decrease in fall off time is a suggestive of the depression of the central nervous system [40]. These data show that μ-TRTX-Hhn1b at a high dose might impede the coordination and balance of rat (motor side effects). Nav1.7 is the most sensitive to μ-TRTX-Hhn1b, but the toxin also inhibits other TTX-S VGSCs in rat DRG cells at a high concentration. Three TTX-S VGSC subtypes, Nav1.1, 1.6 and 1.7, are found in rat DRG cells. We assumed that the inhibition of other subtypes other than Nav1.7 might lead to some side effects [41]. For example, Nav1.6 is widely expressed in the central and peripheral nervous system. A null mutation of the Nav1.6 gene in mice impairs synaptic transmission at neuromuscular junctions and then causes severe paralysis, muscle atrophy and juvenile death [42]. Richard W. Carr and his colleagues confirmed the role of Nav1.6 in mediating the symptoms of oxaliplatin neuropathy [43]. Oxaliplatin neuropathy is characterized by sensory paresthesias and muscle cramps. It is possible that the inhibition of Nav1.6 by the toxin might result in inactivity and paralysis in animals.

## 3. Experimental Section

### 3.1. Peptide Synthesis, Folding and Purification

μ-TRTX-Hhn1b was synthesized starting from a PAL-PEG-PS resin on an automatic peptide synthesizer (PerSeptive Biosystems, Foster City, CA, USA) using an Fmoc/*tert*-butyl strategy and the HOBt/TBTU/NMM coupling method. Peptide synthesis was accomplished on a 0.1-mmol scale. The terminal Fmoc group was removed by treatment with 1:4 piperidine/*N*,*N*-dimethylformamide (v/v). After completion of the synthesis, the peptide was cleaved from the resin with the simultaneous removal of side chain protective groups by treatment with reagent K (82.5% trifluoroacetic acid, 5% double distilled H_2_O, 5% phenol, 5% thioanisole and 2.5% ethanedithiol) for 2 h at room temperature. The resin was then filtered, and the free peptide was precipitated in cold ether at 4 °C. After centrifugation and washing once with cold ether, the peptide was dissolved in 20% acetic acid and lyophilized. The reduced peptides were purified by semipreparative reverse-phase HPLC (Waters 1525 HPLC, Waters Corporation, Milford, UK) using a 45-min linear gradient of 5%–50% eluent B (0.1% trifluoroacetic acid in acetonitrile) in eluent A (0.1% trifluoroacetic acid in double distilled H_2_O) over 45 min on a C_18_ column (Luna, 10 mm × 250 mm) at a 2 mL/min flow rate. Fractions were analyzed by analytical HPLC, and more than 95% pure fractions were pooled and lyophilized. The linear peptides were oxidized with glutathione and purified using the method recently described [44,45,46]. The molecular weights of the reduced peptides or oxidized peptides were checked by matrix-assisted laser desorption/ionization time-of-flight (MALDI-TOF) mass spectrometry (UltraFlex I, Bruker Daltonics). For μ-TRTX-Hhn1b, the measured molecular mass corresponded to the predicted value within 1.0 unit, consistent with the correctness of the sequence and the complete removal of all side chain protection groups.

### 3.2. Electrophysiological Assays

A human Nav1.7 channel plasmid (accession No. NM_002977) and a plasmid for green ﬂuorescent protein were transiently transfected into human embryonic kidney 293 (HEK293) cells using Lipofectamine 2000 (Invitrogen, Carlsbad, CA, USA) according to the manufacturer’s instructions. HEK293 cells were grown under standard tissue culture conditions (5% CO_2_; 37 °C) in DMEM supplemented with 10% FBS. The β_1_ subunit was cotransfected with the Nav1.7 channel to increase the current density. Cells with green ﬂuorescence were selected for whole-cell patch-clamp recording at 36–72 h after transfection. Patch-clamp experiments were performed at room temperature. Suction pipettes (2.0–3.0 MΩ) were made from borosilicate glass capillary tubes using a two-step vertical micropipette puller. The pipette solution contained (in mM): CsCl 145, MgCl_2_∙6H_2_O 4, HEPES 10, EGTA 10, glucose 10, ATP 2 (pH 7.2). The external solution contained (in mM): NaCl 145, KCl 2.5, CaCl_2_ 1.5, MgCl_2_.6H_2_O 1.2, HEPES 10, glucose 10 (pH 7.4). Experimental data were collected and analyzed using the program Pulse/Pulsefit 8.0 (HEKA Electronics, Pfalz, Germany). Macroscopic sodium currents were filtered at 5 kHz and digitized at 20 kHz with an EPC-9 patch-clamp amplifier (HEKA Electronics, Pfalz, Germany). Series resistance was kept near 5 MΩ and compensated to 65%–70%; linear capacitative and leakage currents were digitally subtracted using the P/4 protocol. Dose-response curves were fitted by the Hill equation as follows: y = 1/[1 + 10^(log *EC*_50_ − *x*)^*h*^^], where y is the fraction of current after the application of toxin, EC_50_ is the concentration at half-maximal efficacy, x is the toxin concentration and h is the Hill coefficient. Both the steady-state activation and inactivation curves were fitted by the Boltzmann equation as follows: *G*_*N*av_ / *G*_max_ = 1/(1 + exp^[(*V*_1/2_ − *V*) / *K_m_*]^), where Gmax represents maximal G_Nav_, V_1/2_ is the half-maximal activation voltage, V is the membrane potential and K_m_ is the slope factor.

### 3.3. Animal

The ICR mice (weight 20–25 g/each) and Sprague-Dawley rats (180–220 g/each) used in this study were purchased from the Experimental Animal Center of SLac-kinda (Changsha, China). The animals were maintained at 25–28 °C and allowed access to food (normal laboratory chow) and tap water *ad libitum*. All of the experimental protocols to the animals were approved by the Animal Care and Use Committee (ACUC) at the Hunan Province Animal Management Office (HPAMO). The rats were habituated to the laboratory environment for 3 days before the sensory test. For von Frey assessment, rats were habituated to the laboratory 2 h/day for 2 days before evaluation. Each rat was tested with only one drug and one dose. All animal experiments were performed in a blinded manner.

### 3.4. Abdominal Constriction Response Caused by Acetic Acid

The abdominal constriction responses induced by intraperitoneal (i.p.) injection of 0.2 mL acetic acid (0.8%), including contraction of the abdominal muscle and stretching of the hind limbs, were performed according to procedures described by Collier *et al.* [28]. Mice (male, weighing 20–25 g) were pre-treated with the test samples dissolved in saline by intraperitoneal (i.p.) injection for 15 min prior to acetic acid injection. Control animals received the same volume of vehicle (saline: 0.9% sodium chloride solution). After the challenge, mice were individually placed into open polyvinyl cages (30 cm × 40 cm × 30 cm), and the abdominal constriction responses were counted cumulatively during 30 min, which was represented as the sum of constriction (S). The inhibition of pain responses was calculated by the equation as follows: inhibition of pain responses % = (S_saline_ − S_drug_)/S_saline_ per animal. One hundred percent corresponds to complete reversal of pain, equivalent to the non-injury value, and 0% corresponds to the value from the saline group.

### 3.5. Formalin Test

Acute and chronic nociceptive responses were measured using the rat paw formalin test as descried by Dubission [47] and modified by Malmberg [48]. Adult Sprague-Dawley rats (male, weighing 180–220 g) were used for this behavioral test. During the test, each rat was placed into open polyvinyl cages (30 cm × 40 cm × 30 cm) and at least 30 min before administration of the toxin. All agents were administered 30 min prior to s.c. intraplantar formalin injection. A volume of 50 μL formalin (5%) solution was used as previously described [46] and injected into the plantar surface of one hind paw. Lifting, favoring, licking, shaking and flinching of the injected paw were recorded as nociceptive responses [49]. The total time of the nociceptive response was measured every 5 min and expressed in min (mean ± SEM). Recording of nociceptive behavior commenced immediately after formalin injection and was continued for 40 min.

### 3.6. Spinal Nerve Injury Model

The spared nerve injury was produced as described previously by Decosterd and Woolf [38]. The rats (male, weighing 180–220 g) were anesthetized with ketamine (300 mg/kg) and placed on a pad. Aseptic techniques were used, and the sciatic nerve and its three terminal branches, including the sural, common peroneal and tibial nerves, were exposed, ligated and transected. Muscle and skin were closed with 4-0 polydiaxone and wound clips. Sham controls involved the exposure of the sciatic nerve and its branches without any lesion.

Allodynia was assessed 3 days after SNI surgery, and only rats that developed allodynia, as defined by a significant decrease in their mechanical threshold using von Frey filament, were used. Tactile allodynia was assessed with calibrated von Frey filaments (Stoelting, Wood Dale, IL, USA) using an up-down paradigm. Mechanical sensitivity was determined by applying a series of twenty calibrated von Frey filaments (0.008–2 g, 2–60 g) to the plantar aspect of the left hind paw. Rats that displayed pre-injury baseline measurements <15 g were not included in the study. A response was indicated by brisk withdrawal of the hind paw.

### 3.7. Rotarod Model of Motor Coordination

Motor activity was assessed using a rotarod apparatus for rats (Coulbourn Instruments, Lehigh Valley, PA, USA) [50]. The rotarod might be set at any speed from 0 to 50 rpm. For motor coordination assessment, rats were trained for 2 days by walking them each day on an accelerating rotarod from 4 to 40 rpm for 10 min. Only those animals that demonstrated their ability to remain on the revolving rod for at least four min were used for the test. At the beginning of the rotarod test, trained rats were tested 0 min before and 30 min and 90 min after μ-TRTX-Hhn1b or mexiletine administration. Motor performance was considered as the latency to fall off the rotarod apparatus determined from the mean time in three trials for each rat at each time.

### 3.8. Statistical Analysis

Rats and mice were randomly assigned to each of treated or control groups, and the investigator evaluating the animals was blinded to their treatments. Results were presented as the mean ± SEM. Statistical analysis was carried out by the Student’s *t*-test or a 1-way or 2-way analysis of variance followed by Student–Newman–Keuls’ or Bonferroni posttests when appropriate. *p* < 0.05 was considered to be statistically significant.

## 4. Conclusions

In conclusion, our results demonstrate that μ-TRTX-Hhn1b could dose-dependently reverse hyperalgesia in the abdominal constriction and formalin models of inflammatory pain and allodynia in the SNI model of neuropathic pain. We proposed that the antinociceptive effect of μ-TRTX-Hhn1b was attributed to its ability to inhibit the TTX-S VGSCs, especially Nav1.7, in DRG neurons, thereafter blocking the transduction of the pain pathway. Significantly, our study indicated that, as for the analgesic effects in the three pain models tested, μ-TRTX-Hhn1b might be comparable with or superior to morphine and mexiletine, which are both clinical drugs. It should be noted that abnormal behaviors, such as inactivity and paralysis, were observed in some mice and rats treated with the highest dose of μ-TRTX-Hhn1b. However, no animal death occurred in all tests. It was assumed that the side effects might result from the inhibition of μ-TRTX-Hhn1b on some other VGSC subtypes, such as Nav1.6. Therefore, amino acid mutations will be conducted to enhance the selectivity of μ-TRTX-Hhn1b on Nav1.7, which would be expected to reduce the side effects, but the pain relief effect of the toxin would remain. Taken together, μ-TRTX-Hhn1b efficiently alleviated acute inflammatory pain and chronic neuropathic pain in animals and provided an attractive template for further clinical analgesic drug design.

## References

[1] Catterall W.A. (2012). Voltage-gated sodium channels at 60: Structure, function, and pathophysiology. J. Physiol..

[2] Cummins T.R., Rush A.M. (2007). Voltage-gated sodium channel blockers for the treatment of neuropathic pain. Expert. Rev. Neurother..

[3] Dib-Hajj S.D., Cummins T.R., Black J.A., Waxman S.G. (2010). Sodium channels in normal and pathological pain. Annu. Rev. Neurosci..

[4] Dib-Hajj S.D., Black J.A., Waxman S.G. (2009). Voltage-gated sodium channels: Therapeutic targets for pain. Pain Med..

[5] Wang J., Yarov-Yarovoy V., Kahn R., Gordon D., Gurevitz M., Scheuer T., Gatterall W.A. (2011). Mapping the receptor site for alpha-scorpion toxins on a Na^+^ channel voltage sensor. Proc. Natl. Acad. Sci. USA.

[6] Zhang J.Z., Yarov-Yarovoy V., Scheuer T., Karbat I., Cohen L., Gordon D., Gurevitz M., Catterall W.A. (2011). Structure-function map of the receptor site for b-scorpion toxins in domain II of voltage-gated sodium channels. J. Biol. Chem..

[7] Liu Y., Li D., Wu Z., Li J., Nie D.S., Xiang Y., Liu Z.H. (2012). A positively charged surface patch is important for hainantoxin-IV binding to voltage-gated sodium channels. J. Pept. Sci..

[8] Drenth J.P., Waxman S.G. (2007). Mutations in sodium-channel gene SCN9A cause a spectrum of human genetic pain disorders. J. Clin. Investig..

[9] Fertleman C.R., Baker M.D., Parker K.A., Moffatt S., Elmslie F.V., Abrahamsen B., Ostman J., Klugbauer N., Wood J.N., Gardiner R.M. (2006). SCN9A mutations in paroxysmal extreme pain disorder: Allelic variants underlie distinct channel defects and phenotypes. Neuron.

[10] Cox J.J., Reimann F., Nicholas A.K., Thornton G., Roberts E., Springell K., Karbani G., Jafri H., Mannan J., Raashid Y. (2006). An SCN9A channelopathy causes congenital inability to experience pain. Nature.

[11] Dib-Hajj S.D., Estacion M., Jarecki B.W., Tyrrell L., Fischer T.Z., Lawden M., Cummins T.R., Waxman S.G. (2008). Paroxysmal extreme pain disorder M1627K mutation in human Nav1.7 renders DRG neurons hyperexcitable. Mol. Pain.

[12] Estacion M., Dib-Hajj S.D., Benke P.J., Te Morsche R.H., Eastman E.M., Macala L.J., Drenth J.P., Waxman S.G. (2008). Nav1.7 gain-of-function mutations as a continuum: A1632E displays physiological changes associated with erythromelalgia and paroxysmal extreme pain disorder mutations and produces symptoms of both disorders. J. Neurosci..

[13] Dib-Hajj S.D., Cummins T.R., Black J.A., Waxman S.G. (2007). From genes to pain: Nav1.7 and human pain disorders. Trends Neurosci..

[14] Sheets P.L., Jackson J.O., Waxman S.G., Dib-Hajj S., Cummins T.R. (2007). A Nav1.7 channel mutation associated with hereditary erythromelalgia contributes to neuronal hyperexcitability and displays reduced lidocaine sensitivity. J. Physiol..

[15] Ahmad S., Dahllund L., Eriksson A.B., Hellgren D., Karlsson U., Lund P.E., Meijer I.A., Meury L., Mills T., Moody A. (2007). A stop codon mutation in SCN9A causes lack of pain sensation. Hum. Mol. Genet..

[16] Nilsen K.B., Nicholas A.K., Woods C.G., Mellgren S.I., Nebuchennykh M., Aasly J. (2009). Two novel SCN9A mutations causing insensitivity to pain. Pain.

[17] Cestele S., Catterall W.A. (2000). Molecular mechanisms of neurotoxin action on voltage-gated sodium channels. Biochimie.

[18] Bosmans F., Swartz K.J. (2010). Targeting voltage sensors in sodium channels with spider toxins. Trends Pharmacol. Sci..

[19] Saez N.J., Senff S., Jensen J.E., Er S.Y., Herzig V., Rash L.D., King G.F. (2010). Spider-venom peptides as therapeutics. Toxins.

[20] Billen B., Bosmans F., Tytgat J. (2008). Animal peptides targeting voltage activated sodium channels. Curr. Pharm. Des..

[21] Catterall W.A., Cestèle S., Yarov-Yarovoy V., Yu F.H., Konoki K., Scheuer T. (2007). Voltage-gated ion channels and gating modifier toxins. Toxicon.

[22] Klint J.K., Senff S., Rupasinghe D.B., Er S.Y., Herzig V., Nicholson G.M., King G.F. (2012). Spider-venom peptides that target voltage-gated sodium channels: Pharmacological tools and potential therapeutic leads. Toxicon.

[23] Wood D.L., Miljenović T., Cai S., Raven R.J., Kaas Q., Escoubas P., Herzig V., Wilson D., King G.F. (2009). ArachnoServer: A database of protein toxins from spiders. BMC Genomics.

[24] Norman I.P. (2014). The World Spider Catalog.

[25] Liu Z.H., Dai J., Chen Z.R., Hu W.J., Xiao Y.C., Liang S.P. (2003). Isolation and characterization of hainantoxin-IV, a novel antagonist tetrodotoxinsensitive sodium channels form the Chinese bird spider Ornithoctorus hainana. Cell. Mol. Life Sci..

[26] Li D.L., Xiao Y.C., Xu X., Xiong X., Lu S., Liu Z., Zhu Q., Wang M., Gu X., Liang S. (2004). Structure-activity relationships of hainantoxin-IV and structure determination of active and inactive sodium channel blockers. J. Biol. Chem..

[27] Liu Z.H. (2014).

[28] Collier H.O.J., Dinneen J.C., Johnson C.A., Schneider C. (1968). The abdominal constriction response and its suppression by analgesic drugs in the mouse. Br. J. Pharmacol. Chemother..

[29] Vinegar R., Truax J.F., Selph J.L., Johnston P.R., Vane J.R., Ferreira S.H. (1979). Antagonism of pain and hyperalgesia. Anti-inflammatory Drugs. Handbook of Experimental Pharmacology.

[30] Kumazawa T., Mizumura K., Koda H., Fukusako H. (1996). EP receptor subtypes implicated in the PGE2-induced sensitization of polymodal receptors in response to bradykinin and heat. J. Neurophysiol..

[31] Correa C.R., Kyle D.J., Chakraverty S., Calixto J.B. (1996). Antinociceptive profile of the pseudopeptide B2 bradykinin receptor antagonist NPC 18688 in mice. Br. J. Pharmacol..

[32] Goettl V.M., Larson A.A. (1998). An antagonist of substance P N-terminal fragments, D-substance P(1–7), reveals that both nociceptive and antinociceptive effects are induced by substance P N-terminal activity during noxious chemical stimulation. Brain Res..

[33] Ferrira S.H., Vane S.R. (2005). New aspects on the model of action of nonsteroid anti-inflammatory drugs. Annu. Rev. Pharmacol..

[34] Perianayagam J.B., Sharma S.K., Joseph A., Christina A.J. (2004). Evaluation of antipyretic and analgesic activity of Emblica officinalis Gaertn. J. Ethnopharmacol..

[35] Hong Y., Abbott F.V. (1995). Peripheral opioid modulation of pain and inflammation in the formalin test. Eur. J. Pharmacol..

[36] Moncada S., Ferreira S.H., Vane J.R. (1975). Inhibition of prostaglandin biosynthesis as the mechanism of analgesia of aspirin-like drugs in the dog knee joint. Eur. J. Pharmacol..

[37] Bennett G.J., Xie Y.K. (1988). A peripheral mononeuropathy in rat that produces disorders of pain sensation like those seen in man. Pain.

[38] Decosterd I., Woolf C.J. (2000). Spared nerve injury: An animal model of persistent peripheral neuropathic pain. Pain.

[39] Erichsen H.K., Hao J.X., Xu X.J., Blackburn-Munro G. (2003). A comparison of the antinociceptive effects of voltage-activated Na^+^ channel blockers in two rat models of neuropathic pain. Eur. J. Pharmacol..

[40] Dunham N.W., Miya T.S. (1957). A note on a simple apparatus for detecting neurological deficit in rats and mice. J. Am. Pharm. Assoc..

[41] Goldin A.L. (2011). Resurgence of sodium channel research. Ann. Rev. Physiol..

[42] Burgess D.L., Kohrman D.C., Galt J., Plummer N.W., Jones J.M. (1995). Mutation of a new sodium channel gene, Scn8a, in the mouse mutant “motor endplate disease”. Nat. Genet..

[43] Ruth S., Angelika L., Tobias H., Andrea S.L., Johannes F., Christian A., Peter G., Richard W.C. (2012). Anticancer drug oxaliplatin induces acute cooling-aggravated neuropathy via sodium channel subtype Nav1.6-regurgent and persistent current. Proc. Natl. Acad. Sci..

[44] Zhu Q., Liang S.P., Martin L., Gasparini S., Mènez A., Vita C. (2002). Role of disulfide bonds in folding and activity of leiurotoxin I: Just two disulfides suffice. Biochemistry.

[45] Kabashima T., Yu Z.Q., Tang C.H., Nakagawa Y., Okumura K., Shibata T., Lu J.Z., Masaaki K. (2008). A selective fluorescence reaction forpeptides and chromatographic analysis. Peptides.

[46] Fu C.Y., Xia R.L., Zhang T.F., Lu Y., Zhang S.F., Yu Z.Q., Jin T., Mou X.Z. (2014). Hemokinin-1(4–11)-induced analgesia selectively up-regulates δ-opioid receptor expression in mice. PLoS One.

[47] Dubission D., Dennis S.G. (1977). The formalin tests: A quantitative study of the analgesic effects of morphine, meperidine and brain stem stimulation in rats and cats. Pain.

[48] Malmberg A.B., Yaksh T.L. (1995). Effect of continuous intrathecal infusion of u-conopeptides, N-type calcium channel blockers, on behavior and antinociception in the formalin and hot-plate tests in rats. Pain.

[49] Owoyele V.B., Oloriegbe Y.Y., Balogun E.A., Soladoye A.O. (2005). Analgesic and anti-inflammatory properties of Nelsonia canescens leaf extract. J. Ethnopharmacol..

[50] Rustay N.R., Wahlsten D., Crabbe J.C. (2003). Influence of task parameters on rotarod performance and sensitivity to ethanol in mice. Behav. Brain Res..

